# Alternative splicing of synuclein gamma in endometrial cancer: identification of a novel isoform

**DOI:** 10.18632/oncotarget.4155

**Published:** 2015-06-05

**Authors:** Kathrin Schaal, Marc Hirschfeld, Peter Bronsert, Hannah Füllgraf, Markus Jäger, Bettina Herde, Claudia Nöthling, Sebastian Mayer, Thalia Erbes, Elmar Stickeler

**Affiliations:** ^1^ Department of Obstetrics and Gynecology, University Medical Center, Freiburg, Germany; ^2^ Department of Urology, University Medical Center, Freiburg, Germany; ^3^ German Cancer Consortium (DKTK), Freiburg, Germany; ^4^ German Cancer Research Center (DKZF), Heidelberg, Germany; ^5^ Institute of Surgical Pathology, University Medical Center, Freiburg, Freiburg, Germany; ^6^ Comprehensive Cancer Center, University Medical Center, Freiburg, Germany

**Keywords:** synuclein gamma, SNCG, alternative splicing, endometrial cancer, biomarker

## Abstract

Synuclein gamma (SNCG) is under consideration as a potential biomarker in cancer biology. Up to date four different SNCG variants are described. Due to growing evidence suggesting correlations between aberrant alternative splicing processes and cancer progression, we investigated the effects of peritumoural conditions on expression pattern of SNCG in endometrial cancer (EC) *in vitro*. Compared to breast cancer cell lines, mRNA expression levels of all known SNCG isoforms 1–4 are significantly reduced in EC cell lines. We identified a novel alternatively spliced variant of isoform 2 (isoform 2 *short*) which is found highly expressed in EC cell lines. Hypoxia and acidosis trigger an up-regulation of isoform 2 *short*. EC cell lines are characterized by low SNCG protein levels under control conditions, but exhibit a significant increase triggered by hypoxia and acidosis. In addition we analysed the potential association between SNCG protein expression and clinico-pathological parameters in human EC samples. Our findings indicate a grade-dependent induction of SNCG protein expression in endometrial cancer.

We identified for the first time a novel isoform of SNCG that is found specifically expressed in EC. Our results also strongly indicate the existence of a corresponding protein of isoform 2 *short* that potentially plays a critical role in EC cancer progression.

## INTRODUCTION

Endometrial cancer (EC) is with 320.000 newly registered cases yearly, the most common gynaecologic malignancy in developed countries. With approximately 76.000 EC-related registered annual deaths, EC is also the seventh most common cause of death from cancer in women [1]. Endometrial malignancies are a heterogeneous group of tumours which can be histopathologically subdivided into Type 1 or estrogen-dependent endometroid endometrial carcinomas (EEC) and Type 2 or estrogen-independent non-endometroid carcinomas (NEEC) [2]. Those groups differ in aetiology, risk profile, response and prognosis. Type 1 or EECs express estrogen (ER) and progesterone (PR) receptors and are the most common type of sporadic EC. Patients diagnosed with EEC have an excellent prognosis of 83% 5-year-survival [3].

Type 2 tumours or NEECs are usually negative or weakly positive for steroid hormone receptors. While this group represents a minority of total endometrial carcinoma cases, NEECs are usually high-grade tumours with a more aggressive clinical course, poor response rates to chemotherapy and declined outcome. Multitudinous biomarkers such as estrogen/progesterone receptors, β-catenin, Her2/neu, K-ras or p53 were shown to predict prognosis in women with EC [4].

Synuclein gamma (SNCG), also known as breast cancer specific gene 1 (BCSG1), is localized to chromosome 10q23 and is composed of five exons. Four different isoforms were described by now, but only the isoforms 1 und 2 encode for two similar polypeptides with a length of 127 and 126 amino acids, respectively, with a molecular weight of 17 kDa [5–7]. Functionally, SNCG acts as a chaperone protein and was found highly expressed in multiple cancer types such as advanced stage breast and ovarian cancers [8–10]. It has been shown to promote cell growth, increased cell motility and inhibition of stress- and chemotherapy induced apoptosis. SNCG influences several signaling pathways via an activating or blocking function, e.g. extracellular-signal regulated kinase 1/2 (ERK1/2), c-Jun N-terminal kinase 1 (JNK1) or BUB1 (budding uninhibited by benzimidazoles 1) mitotic checkpoint serine/threonine kinase B (BubR1) [11–13]. *In vitro* studies showed that SNCG stimulates membrane-initiated estrogen signaling by chaperoning estrogen receptor alpha 36 (ERα36). SNCG enhances the high-affinity ligand-binding state of ERα36 and significantly stimulates the transcriptional activity of ERα and ligand-dependent mammary tumourigenesis [14–16]. Although the vast majority of EC are characterized by estrogen dependency, only little is known about the functional implications of SNCG in EC so far. Two recent studies identified SNCG overexpression in EC specimen with 20% or 48.3%, respectively, while percentages of about 38% were found in breast cancer [17–19].

Hypoxia and extracellular acidosis, as typical epiphenomena of solid tumours, are important inducers for transcriptional cascades promoting aggressive cancer phenotypes [20]. In recent studies we described changes in alternative splicing pattern of cancer related genes, e.g. Cyr61, and alterations in splicing factor expression pattern induced by altered peritumoural conditions *in vitro* [21]. Hitherto four SNCG isoforms were described. However, detailed analyses on expression pattern remain pending so far.

Due to growing evidence suggesting correlations between aberrant splicing processes and cancer progression, we pursued our present study on the effects of peritumoural conditions on expression pattern of SNCG in EC *in vitro*. We identified a novel isoform of SNCG characterized by up-regulated expression levels under hypoxic and acidic conditions. In accordance to the mRNA results, a corresponding increase in total SNCG protein expression was determined. Thus, we postulate the existence of a functional corresponding protein of the novel SNCG isoform which potentially exerts functional impacts on tumour progression in endometrial malignancies.

## RESULTS

### Marginal SNCG isoform expression in endometrial cancer cells

Since breast cancer cell lines are characterized by high SNCG expression levels we used the previously described SNCG-positive T47D cells as *in vitro* model for SNCG expression monitoring in all experimental approaches. Specific combinatory primer pairs were utilized to detect the expression levels of all distinct SNCG isoforms in four endometrial cancer cell lines. PCR analyses in triplicates revealed uniform low expression levels of all known SNCG variants among EC cell lines tested (supplemental data). In analogy, quantitative real time PCR analysis of the protein-coding isoforms 1 and 2 showed significantly reduced SNCG expression levels in EC cell lines compared to breast cancer cell line T47D.

### Impact of microenvironmental alterations on SNCG splicing pattern

Hypoxia and acidosis are typical peritumoural conditions known to influence splicing pattern of several cancer-related genes. The potential regulatory impact of mimicked tumourbiological microenvironment on splicing pattern of SNCG was analysed in functional cell culture experiments. Cell lines were incubated under hypoxic, acidic and control conditions in parallel and expression levels of all known SNCG mRNA isoforms were investigated by conventional PCR and quantitative real time PCR. Since endometrial cancer cells demonstrated marginal overall SNCG expression levels only -compared to those of highly SNCG-positive T47D control – hypoxia- and acidosis-dependent aberrations in SNCG levels were restricted to mere tendencies (supplemental data).

### SNCG protein expression under hypoxia and acidosis

**Figure 1:**
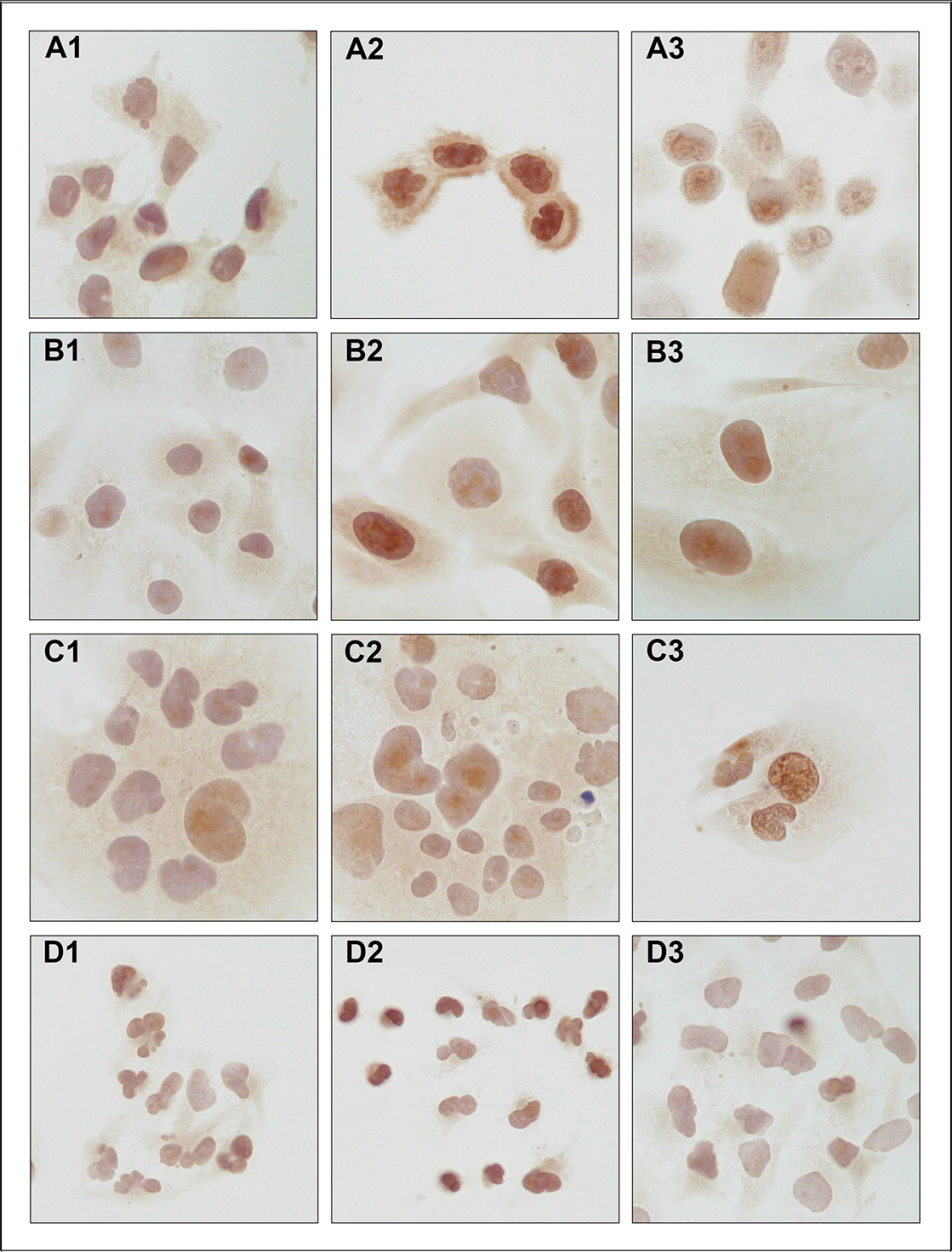
Immunocytochemical detection of SNCG protein expression in endometrial cancer cell lines **A.** MFE-296, **B.** EFE-184, **C.** Ishikawa and **D.** An3-Ca under (1) control conditions, (2) 18 hrs hypoxia (O_2_ > 1%) and (3) extracellular acidosis (pH 6.2). SNCG protein expression under control conditions is marginal and concentrates on perinuclear compartments. Hypoxia and acidosis induce an increase in nuclear and cytoplasmic SNCG protein expression levels. NEEC cell line An3-Ca is characterized by lack of cytoplasmic SNCG protein expression and low nuclear expression under all conditions tested. Immunocytochemistry, triplicate experiments. SNCG antibody sc-10698 (SCBT); counterstained with hemalaun. Magnification x400.

**Figure 2:**
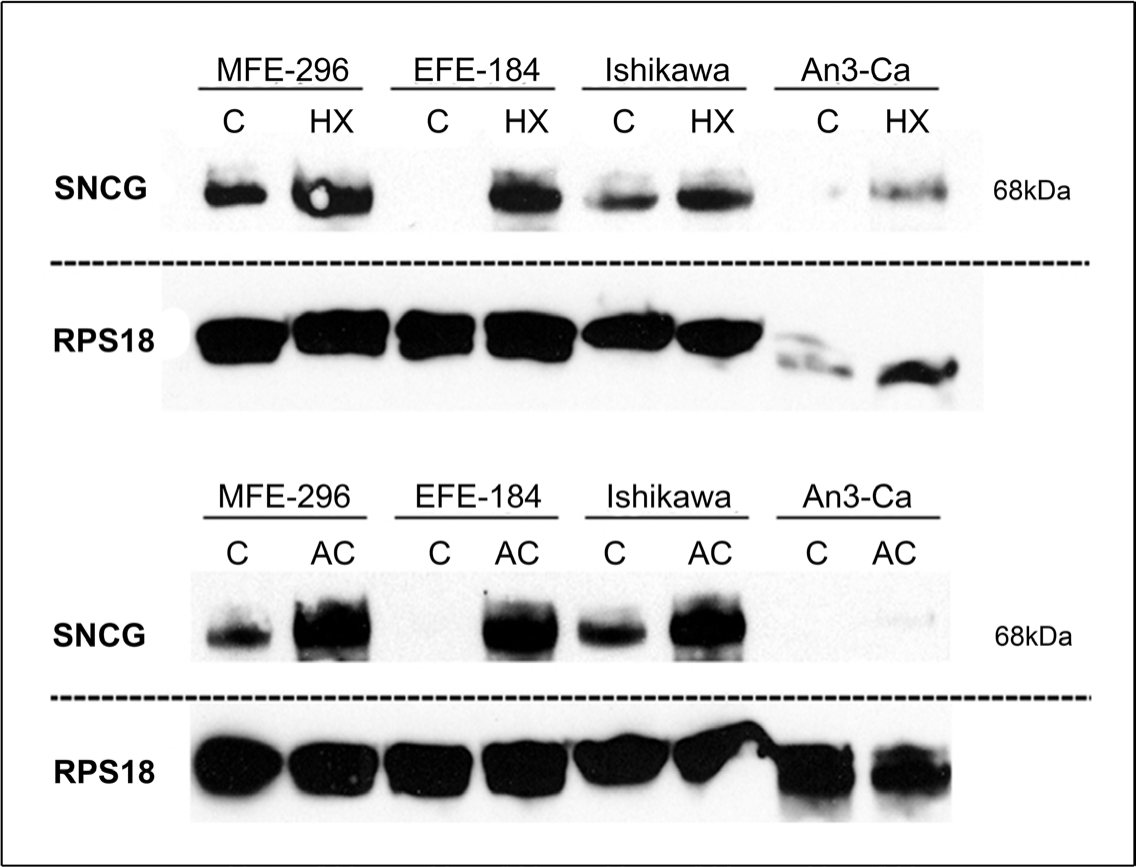
Quantitative detection of SNCG protein Comparison of endometrial cancer cell lines cultured under (C) control conditions versus (HX) hypoxia (18 hrs, O_2_ > 1%) or (AC) acidosis (18 hrs, pH 6.2). Both, hypoxia and acidosis trigger an up-regulation in SNCG protein expression. NEEC cell line An3-Ca demonstrates only marginal SNCG protein expression. RPS18 expression serves as comparative value. *Dashed lines indicate origin from different gels*. SNCG antibody sc-135676 (SCBT) detects tetramer confirmation of SNCG protein (68 kDa). Western blot, triplicate experiments.

SNCG protein expression was determined by immunocytochemical analysis in cell lines treated with hypoxia or extracellular acidosis compared to cells cultured under control conditions. Both, hypoxia and acidosis triggered an increase in nuclear and cytoplasmic SNCG protein levels (Figure 1). In analogy, Western blot analyses showed low SNCG protein expression under control conditions. Hypoxia and acidosis lead to a marked up-regulation in SNCG protein expression (Figure 2). Exceptionally, the ER-negative cell line An3-Ca showed very low overall SNCG protein expression, independent from microenvironmental conditions.

### Novel SNCG isoform

**Figure 3:**
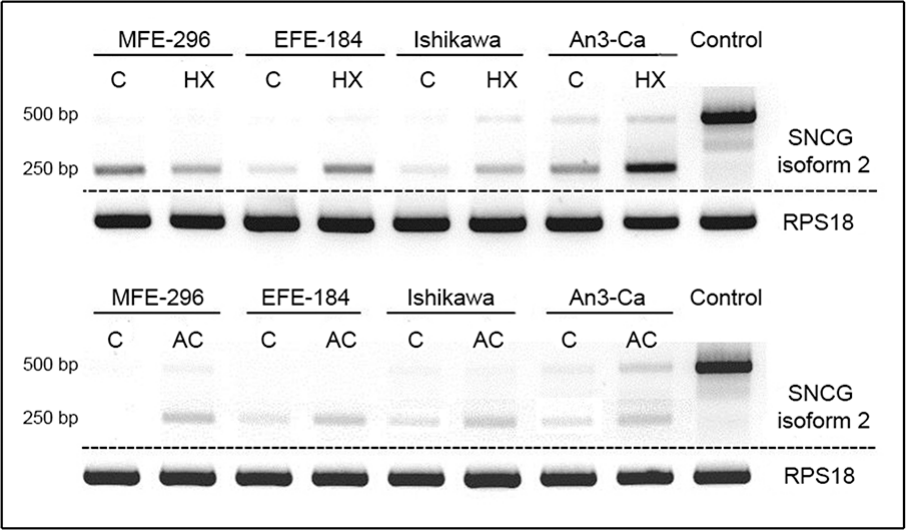
Expression of SNCG isoform 2 in EC cell lines MFE-296, EFE-184, Ishikawa, and An3-Ca under (C) control conditions, (HX) hypoxia (18 hrs, O_2_ > 1%) or (AC) acidosis (18 hrs, pH 6.2) T47D breast cancer cells served as SNCG positive control. SNCG mRNA expression of the expected 500 bp amplicon coding for isoform 2 is not or only marginal detectable in all tested EC cell lines compared to breast cancer cell line T47D. In contrast, a novel 250 bp shorter amplicon demonstrates exclusive expression in EC cell lines. Hypoxia and acidosis trigger an increase in expression levels of the novel splice variant. RPS18 expression serves as a comparative value. *Dashed lines indicate origin from different gels*. PCR.

In addition to the expected SNCG isoform 2 amplicon (505 bp) an approximately 250 bp shorter PCR product occurred in PCR analysis utilizing the specific primer pair for isoform 2 detection. Interestingly, this mRNA variant was detectable in all endometrial cancer cell lines, but not in breast cancer cell line T47D (Figure 3).

On the basis of repeated sequence analysis of the so far unknown amplicon we were able to verify the existence of a novel variant of isoform 2 that is characterized by a 246 bp deletion (from position 355 to 601) within the regular isoform 2 exonic sequence.

**Figure 4:**
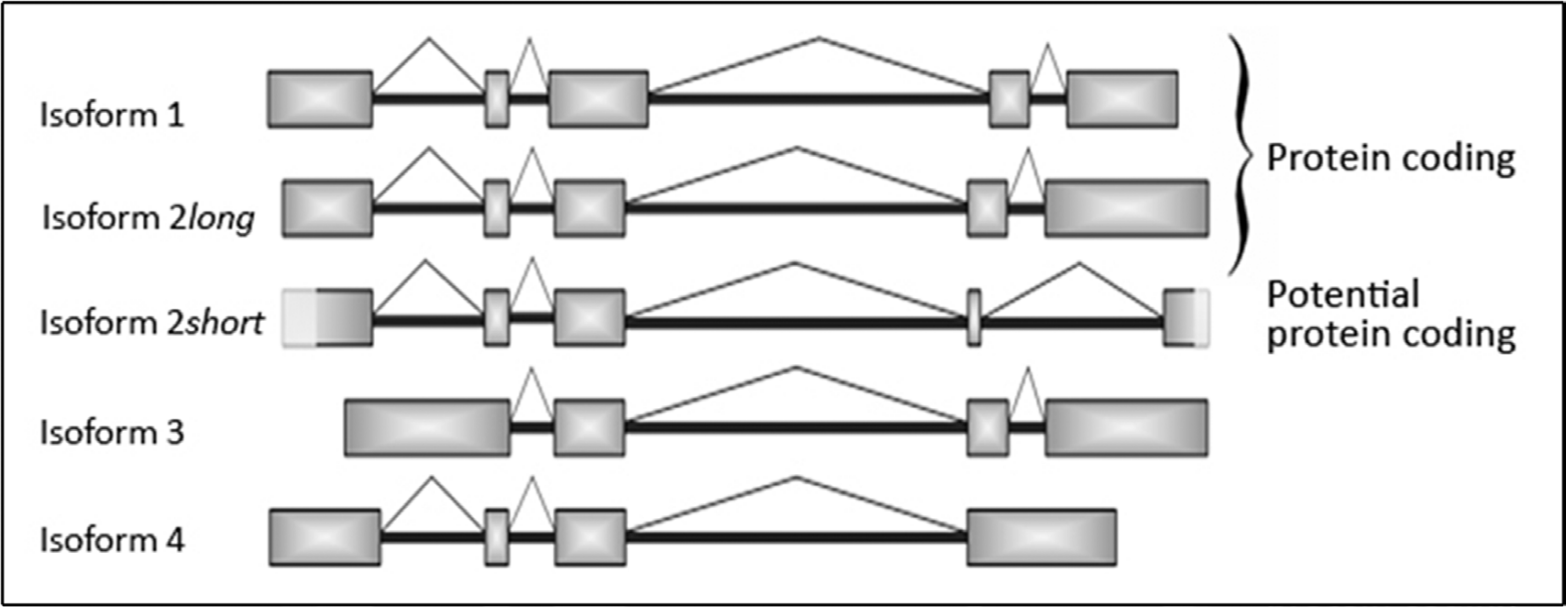
Schematic illustration of the different SNCG mRNA isoforms including novel isoform 2 *short* Newly detected 246 bp shorter isoform 2 *short* is characterized by partial loss of exons 4 and 5. Light grey boxes at the beginning of exon 1 and at the ending of exon 5 highlight sequence parts of the novel mRNA splicing variant isoform 2 *short* that were not verified by sequencing analysis so far.

**Figure 5:**
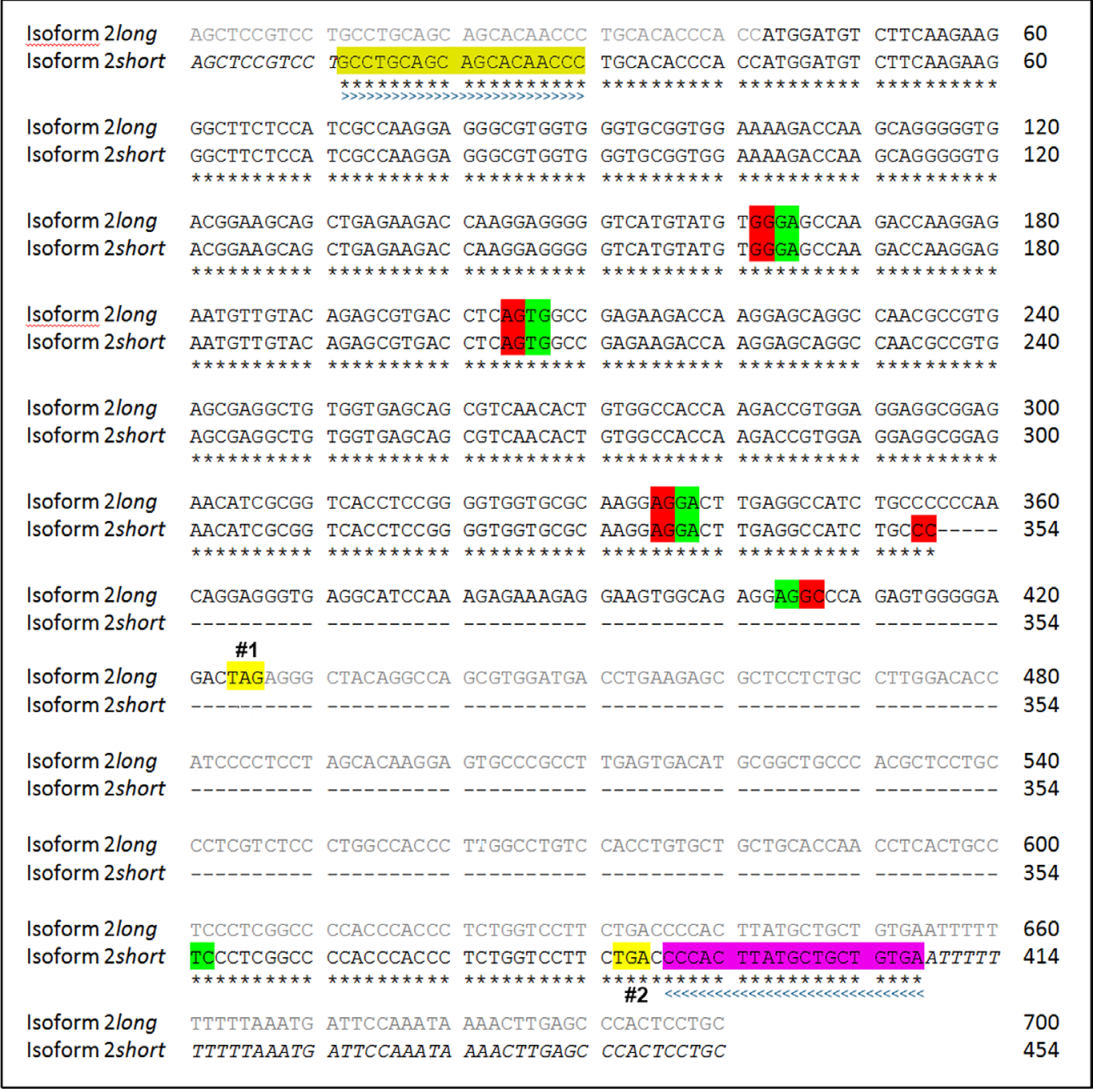
Sequence alignment of SNCG mRNA isoforms 2 long and 2 *short* Isoform 2 long: Sequence 700 bp; 159 A; 208 C; 213 G; 120 T. Isoform 2 *short:* Sequence 454 bp; 108 A; 126 C; 140 G; 80 T. Nucleotides from position 355 to 601 of isoform 2 long are skipped in the newly detected isoform 2 *short*. Below the sequence is a key denoting concordant nucleotides that account for corresponding amino acids (*). Not yet verified nucleotides are shown in italic types. UTR of isoform 2 long is indicated by greyish font colour. Colored boxes indicate the sequence of the used sense (light green, > > >) and antisense (purple, < < < <) primer pair. Beginnings of exons are indicated by green boxes. Endings of exons are indicated by red boxes. Spaces were introduced in order to show skipped nucleotides. (Premature) termination codons/stop codons are highlighted in yellow (#1: isoform 2 long at position 424; #2: isoform 2 short at position 386). Sequence data based on repeated analysis performed by GATC.

This novel isoform, isoform 2 *short*, emerges from a partial skipping of nucleotide sequence spanning from exon 4 to 5 (Figure 4). Via frameshift of open reading frame (ORF) skipping bears the potential to create the occurrence of premature stop codons (PTCs) within the aberrant mRNA, which could consecutively lead either to mRNA degradation by a mechanism called nonsense-mediated decay (NMD) or to a truncated protein isoform 2. Since sequence analyses revealed the loss of the original stop codon of isoform 2, but the introduction of a new stop codon only a few bases downstream of the deletion site (Figure 5). Our findings highly suggest the existence of a corresponding functional truncated protein isoform “2 *short”*. Our repeated attempts to insert full-length isoform 2 *short* into pCMV Script expression vector remained unsuccessful. Up to date no SNCG isoform-specific antibody is commercially available and the supplier of the antibodies utilized in our experiments does not provide detailed information in regard to binding site or epitope.

**Figure 6:**
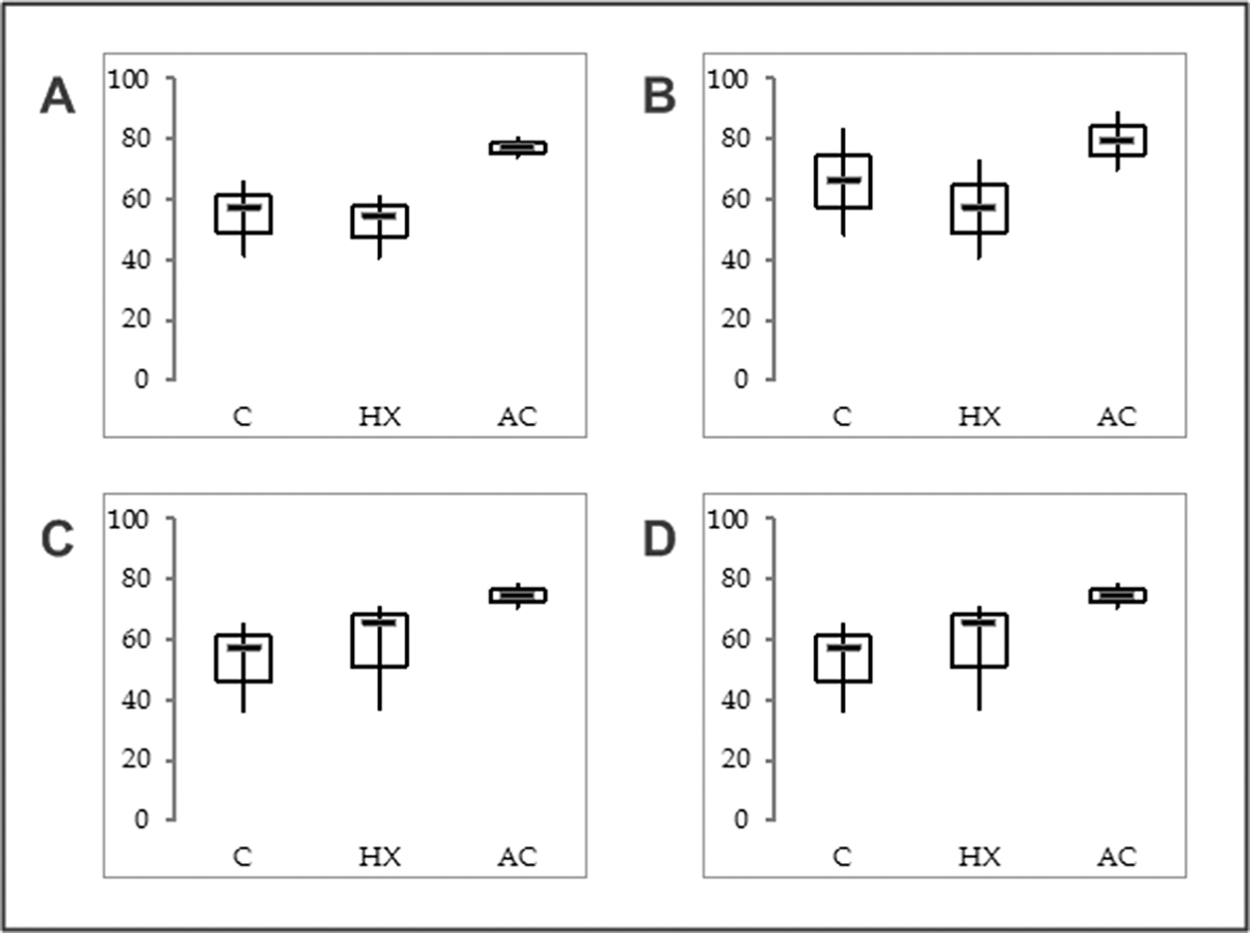
mRNA expression levels of SNCG isoform 2 short in EC cell lines **A.** MFE-296, **B.** EFE-184, **C.** Ishikawa and **D.** An3-Ca under (C) control conditions, (HX) hypoxia (18 hrs, O_2_ > 1%) or (AC) acidosis (18 hrs, pH 6.2). RPS18 mRNA expression served as comparative value (100%). Box plots based on triplicate qPCR analysis. *Thick lines, median (50% percentile); grey boxes, 25% to 75% percentile; thin lines, minimal and maximal value*.

Interestingly, only the novel isoform 2 *short* could be characterized by alterations in mRNA expression compared to the other investigated known SNCG isoforms when cultured under hypoxic and acidic conditions. Both peritumoural conditions lead to an increased expression of isoform 2 *short* mRNA. We verified our findings using a specific primer pair for isoform 2 *short* in quantitative real time PCR analyses (Figure 6).

### SNCG expression in endometrial cancer tissue

**Figure 7:**
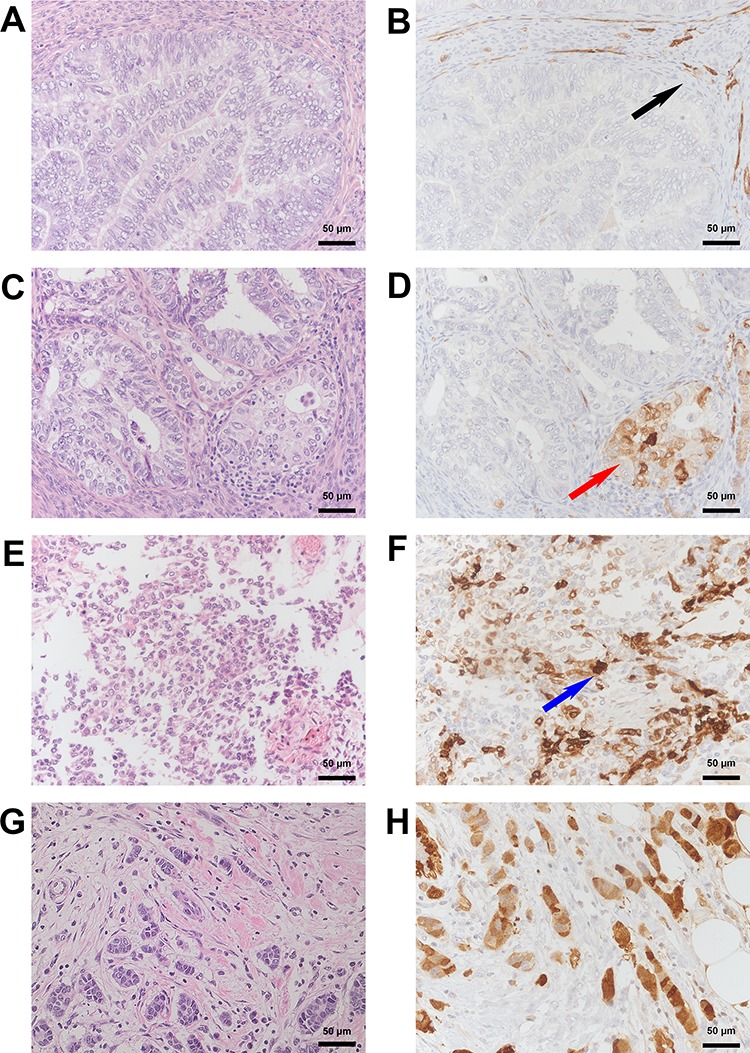
Immunohistochemical detection of SNCG protein in endometrial and breast cancer tissue **A.** HE and corresponding **B.** SNCG negative immunohistological staining from a well differentiated endometroid cancer. Note the presence of SNCG in the surrounding smooth muscle (*black arrow*). **C.** HE and corresponding **D.** SNCG cytoplasmatic positive immunohistological staining (*red arrow*) from a moderately differentiated endometroid cancer. **E.** HE and corresponding **F.** SNCG positive immunohistological staining (*blue arrow*) from a poorly differentiated endometroid cancer with a sarcomatoid component. **G.** HE and corresponding **H.** SNCG positive control for immunohistological staining from breast tumour tissue for both, cytoplasmatic and nuclear staining. A, C, E, G: HE Staining; B, D, F, H: SNCG antibody sc-10698 (SCBT); counterstained with hemalaun. Magnification x200.

A pilot set of eleven randomly selected samples of EC tissue was examined to investigate the expression and localization pattern of SNCG protein under pathologic conditions. Immunohistochemical detection of intracellular SNCG protein revealed predominant perinuclear localization and weak expression in cytoplasm. Strongest staining pattern became apparent in poorly differentiated grade 3 tumours. Moderate SNCG protein expression was found in grade 2 tumours, while no protein signal could be detected in low grade EC tumour samples. Unspecific staining pattern characterized portions of smooth muscle tissue. Breast cancer tissue served as SNCG positive control in this analysis and showed highest SNCG protein expression as expected (Figure 7).

## DISCUSSION

Identification and cancer management of risk patients relies on robust biomarkers. The present models for prediction of prognosis and treatment response has its limitations. In contrast to other tumour entities like breast or colon cancer, a molecular classification of EC is not widely and routinely implemented in clinical practice [22]. Novel molecular biomarkers to assist clinical decision making are needed.

Aberrant SNCG expression was shown associated with advanced tumour stages in breast cancer [8–10]. Increased levels of SNCG protein expression reduce apoptosis and induce tumour growth [12, 14, 23]. Our investigations showed markedly increased protein expression of Synuclein gamma in EC under hypoxic and acidic conditions. Those conditions are well-known microenvironmental epiphenomena of solid tumours and can act as inductors of transcriptional cascades regulating tumour growth [20]. Our results demonstrate the tumourbiological importance of SNCG protein expression under these cellular stress conditions. High levels of SNCG expression seem to provide certain selection advantages for malignant cells. However, this protein expression was not found allegeable by the mRNA expression of the so far known protein-coding SNCG isoforms 1 and 2.

Alternative splicing exerts a key role in gene expression regulation. The process largely contributes to a wider diversity in protein architecture and supports biologic complexity. More than 90% of human genes encode splice isoforms, some of which even trigger antagonistic functions. Especially tumour cells are known profiteers from this adaptive ability of creating alternative protein isoforms under varying cellular conditions [24–26]. Research on new molecular biomarkers must include all alternative spliced isoforms of one target gene. It is crucial to know the global expression profile of oncogenes in cancer cells for predicting prognosis and developing novel therapeutic strategies for malignancies.

The striking difference in all studies on SNCG published to date, was the disregard of investigation of mRNA expression of the four distinct SNCG isoforms. Unspecific primer pairs amplifying several isoforms of SNCG at once were used in past studies [27–29].

In our investigations we used isoform-specific primer pairs to identify the expression profile of the different protein- and non-protein-coding isoforms.

We were able to identify a novel alternative spliced isoform of SNCG. This isoform is a variant of the protein-coding isoform 2 lacking 246 consecutive nucleotides within exons 4 and 5. The novel isoform showed the highest levels of mRNA expression of all known SNCG isoforms in the exhibited EC lines tested. Sequence analyses revealed that the stop codon of the regular isoform 2 long on position 424 is missing in isoform 2 *short*. However, a new stop codon is introduced on position 386 of isoform 2 *short* (Figure 5). By mischance, additional investigations verifying whether this newly identified SNCG isoform encodes for a functional protein failed, because there was no information on binding sides for all commercially available antibodies against SNCG, respectively.

Most interestingly, our correlative data on mRNA expression levels account for the existence of a corresponding functional protein of isoform 2 *short* in all likelihood. The marked expression alterations in SNCG expression levels determined in EC cells in Western blot and immunochemistry cannot be set in relation to the mRNA data of the known established isoforms 1 and 2. The newly discovered SNCG isoform 2 *short* showed the highest levels of mRNA expression compared to all known SCNG isoforms in EC *in vitro*. Furthermore our data demonstrate that isoform 2 *short* is clearly influenced by acidosis and hypoxia in contrast to the other analysed isoforms 1–4. Hence, it is very likely that this novel variant accounts for the high protein expression levels and changes in expression pattern triggered by peritumoural conditions. We have no information about the RNA sequence up- and downstream of our primers. However, those parts of the RNA sequence are very similar in all known SNCG isoforms. We suppose that there is no difference to isoform 2 *long*.

The detected alterations in alternative splicing pattern of SNCG isoforms in EC cell lines compared to breast cancer line T47D suggest a different functional role of SNCG in these two malignancies, respectively. The existence of a corresponding protein isoform 2 *short* is very likely since our results revealed high expression of the newly detected isoform 2 *short* in the different EC cell lines compared to the low mRNA expression of the so far known isoforms 1–4. The functional role of isoform 2 *short* in cancer cells is not clarified yet. However, we hypothesize, that this isoform encodes for a biologically functional protein.

In accordance to previous studies on human EC samples we also found a positive correlation of SNCG-immune protein expression and tumor grade. The immunohistochemical analysis of a larger EC tissue cohort (>100 samples) is currently projected. Despite those findings, our *in vitro* studies did not account for any correlations between SNCG protein expression and aggressive cell type behaviour [18, 28]. Actually, the lowest SNCG RNA isoform levels and corresponding protein expression were identified in NEEC cell line An3-Ca. Since *in vitro* models allow limited validity only in regard to tumour tissue biology, clinical studies with specific antibodies against distinct SNCG protein isoforms are needed to elucidate the tumourbiological relevance of SNCG in aggressive NEECs compared to EECs.

## CONCLUSIONS

In conclusion, our data account for an aberrant functional impact of SNCG in EC, compared to breast or other cancers. We identified a novel alternatively spliced isoform of SNCG, isoform 2 *short,* which demonstrated specific expression prevalence for EC. While only low SNCG protein expression was detectable under regular culture conditions, Western blot and immunocytochemical analysis showed induction of SNCG protein expression triggered by hypoxia and acidosis. The detected corresponding protein levels were not allegeable by the mRNA levels of the so far known SNCG isoforms 1–4. However, the newly identified isoform 2 *short* accounts for protein expression increase in all likelihood, therefore bearing an oncogenic potential in EC.

Further experiments with isoform-specific antibodies are needed to verify the existence of a corresponding protein of isoform 2 *short* mRNA.

## MATERIALS AND METHODS

### Cell culture and transient transfection

Cultured in a humidified incubator (37°C, 5% CO_2_, 95% air), the established human breast cancer cell line T47D (ii) and endometrial cancer cell lines MFE-296 (i), EFE-184 (ii), Ishikawa (i) and An3-Ca (ii) were maintained in either (i) Dulbecco's modified Eagle medium (DMEM) supplemented with 10% newborn bovine serum (PAA Laboratories GmbH, Pasching, Austria), 1% of 1 mol/L HEPES buffer (Life Technologies™, Thermo Fischer Scientific Inc., Darmstadt, Germany) and 100 U/ml penicillin/streptomycin (PAA) or (ii) Quantum263 (PAA) medium supplemented with 100 U/ml penicillin/streptomycin (PAA).

For hypoxia experiments the cell lines were transferred to a hypoxic chamber with oxygen < 1% and placed in the same incubator. For acidosis experiments culture media were supplemented with lactic acid (0.2%, pH 6.2, Sigma-Aldrich^®^, Taufkirchen, Germany). Cells were cultured in parallel under hypoxic, acidic and under control conditions for 18 hrs, followed by immediate RNA extraction. For immunocytochemical detection (ICC) of SNCG protein all cell lines were cultured similarly under control, hypoxic and acidic conditions on culture slides for 18 hrs. ICC experiments were concluded by immediate fixation of cells for further analysis.

### RNA extraction and PCR analyses

Isolation of total RNA from cultured cells was performed using TRIzol^®^ Reagent (Life Technologies™, Thermo) according to manufacturer's protocol.

Determined by optical densitometry, 1 μg of purified RNA from each sample was transcribed to cDNA using Maxima Reverse Transkriptase (Thermo), RNAse inhibitor (Life Technologies™, Thermo) and random hexamer primers (New England BioLabs^®^Inc., Ipswich, USA). Contamination with genomic DNA was prevented using Dpn1 enzyme (Life Technologies™, Thermo) for digestion (30 min, 37°C).

The expression profiles of genes of interest were determined by quantitative real time PCR and conventional PCR. CDNA was used as template and conventional PCR using Perpetual Taq DNA polymerase (Roboklon, Berlin, Germany) and specific primers (Apara Bioscience GmbH, Denzlingen, Germany) for isoform 2 (forward primer 5′-GGT CAT GTA TGT GGG AGC C-3′; reverse primer 5′-TCA CAG CAG CAT AAG TGG G’-3), isoform 3 ( forward primer 5′-CAG ACA CAG CAG GAA GAG G-3′; reverse primer 5′-GGA CTT GAG GCC ATC TGC-3′), isoform 4 (forward primer 5‘-CTC GAG CCA GCT CAA GC-3′**;** reverse primer 5′-GCA GAT GGC CTC AAG TCC-3′) and *RPS18* RNA as internal control/housekeeping gene (forward primer 5′-AAC TCA CTG AAG ATG AGG TG-3′; reverse primer, 5′-CAG ACA AGG CCT ACA GAC TT-3′). For quantitative real time PCR specific primers (Apara) were used for isoform 1 (forward primer 5′-CAC GGT CTT GGT GGC CAC-3′; reverse primer 5′-CCA GCC AGT GTC CTC CCA TAG-3′), isoform 2 long (forward primer 5′-CAA CAG GAG GGT GAG GCA TCC-3′; reverse primer 5‘-GTG GAG GAG GCG GAG AAC ATC-3‘), isoform 2 *short* (forward primer 5′-CCG TGA GCG AGG CTG TGG TG-3′; reverse primer 5′-GCC GAG GGA GGG CAG TGG-3‘) and RPS18 as internal control (forward primer 5′-AAC TCA CTG AGG ATG AGG TG-3′; reverse primer 5′-CAG ACA AGG CCT ACA GAC TT-3′). All quantitative real time PCR and conventional PCR analyses were performed in triplicates and normalized using corresponding RPS18 RNA expression as a comparative value.

### Sequencing and recombinant protein expression

All SNCG gene identification sequence data is based on http://ensemble.org with gene accession number: ENSG00000173267. Unidentified PCR amplicons were verified by DNA sequencing. The specific novel cDNA was cloned into pJET1.2/blunt Cloning Vector using Clone Jet Kit (Thermo) according to manufacturer's protocol. Commercial DNA sequencing was carried out by GATC biotech (European Genome and Diagnostics Centre, Konstanz, Germany). For recombinant protein expression of isoform 2 *short* specific primers binding at the beginning of exon 1 (forward primer 5′-GCC TGC AGC AGC ACA ACC-3′) or at the end of exon 5 (reverse primer 5′-TTC TCG AGC AGG AGT GGG CTC AAG T-3′), respectively, were used to create full-length cDNA samples of the isoform. CDNA samples were used as templates to clone isoform 2 *short* sequence into the expression vector pCMV Script Vector (Agilent Technologies, Stratagene^®^, Santa Clara, USA). EcR V and Xho I were used as cloning sites. Vector inserts were verified using T3 promotor primers Vector (Agilent Technologies, Stratagene^®^) and insert specific primers (Apara).

### Protein extraction and western blot

Proteins from treated cells were isolated in parallel to RNA extraction using TRIzol^®^ Reagent (Life Technologies™, Thermo) following the manufacturer's protocol. Purified proteins were stored at −80°C for further analyses. Protein concentrations were determined by the bicinchoninic acid assay method. Western blot was performed following the technique of Schagger and von Jagow [30]. Immunodetection was carried out by application of a polyclonal rabbit IgG antibody raised against the SNCG protein (Anti-gamma-Synuclein B-21, sc-135676, Santa Cruz Biotechnology Inc. (SCBT), Dallas, USA) and a peroxidase conjugated secondary anti-rabbit antibody (SCBT). A polyclonal rabbit IgG antibody raised against RPS18 protein (Abcam^®^, Cambridge, UK) and a peroxydase conjugated secondary anti-rabbit (SCBT) antibody was used as loading control. Visualization was performed by enhanced chemiluminescence detection Kit (Life Technologies™, Thermo) and exposure to X-ray film (FUJIFILM Europe GmbH, Duesseldorf, Germany).

### Immunocytochemistry

Cell lines MFE-296, EFE-184, ISHIKAWA and An3-Ca were fixed after experimental treatment using ROTI^®^ Histofix formaldehyde fixation reagent (4% formaldehyde in PBS, pH 7, Carl Roth, Karlsruhe, Germany). Anti-gamma-Synuclein B-21 rabbit antibody (sc-135676, SCBT) was used for detection of SNCG protein. For visualization of SNCG protein expression indirect immunoperoxidase technique was applied. Antigen retrieval was performed in a 10 mmol/L sodium citrate buffer (pH 6.0) followed by inhibition of endogenous peroxidase by incubation for 30 min with 3% H_2_O_2_. Endogenous avidin–biotin was blocked by the use of a commercial biotin blocking system (Agilent Technologies, DAKO, Santa Clara, USA) for 10 min. Unspecific staining was avoided by incubation for 30 min in skimmed milk blocking buffer. Following overnight incubation with SNCG antibody (1:2000) at RT and two washing steps with PBS, slides were exposed to biotinylated anti-rabbit immunoglobulins for 60 min at RT and treated with streptavidin-peroxidase (Agilent Technologies, DAKO, Santa Clara, USA). Staining of SNCG protein was achieved by 3, 3-diaminobenzidine (DAB; Vector Laboratories Inc., Burlingame, USA) and the cells were counterstained with hemalaun (Merck, Darmstadt, Germany).

### Patients and tissue

From a retrospectively maintained tissue-database, eleven randomly selected tissue specimens for endometrial cancer and one for breast cancer (control) were included. Ethical approval was obtained from the ethics committee of the University Medical Center Freiburg.

### Immunohistochemistry

From retrospectively collected FFPE endometrial cancer cohort, tissue specimens of three patients with endometrial cancer were stained for SNCG protein. Tissue specimens were cut into 3 μm thick slices using the Leica RM2255 Microtome. Antigen retrieval was performed at 95°C for a period of 40 minutes in pH 6.1 using DAKO antigen retrieval buffer S1699 (DAKO). Endogenous peroxidase was blocked using H_2_O_2_-Block FlexPeroxidase (EnVision^TM^, DAKO, Lot: 20011164) for 10 min at room temperature. Incubation with Anti-gamma-Synuclein B-21 rabbit antibody (sc-135676, SCBT, 1:100) at room temperature for 30 minutes was followed by staining with a second Antibody Flex Rabbit Linker (K8019, DAKO, Lot: 20010247). The reaction was visualized using FlexHRP (DAKO, LOT: 20011210) for 20 min and visualized using FlexDAB-Chromogen (DAKO) for 10 min. After washing using water, all slides were counterstained with hematoxylin, dehydrated in ascending alcohol concentrations and covered with TissueTek 4770 Coverslipping Film (Sakura^®^, Sakura Finetek Europe B.V., Alphen aan den Rijn, The Netherlands) on TissueTek SCA (Sakura).

### Assessment of SNCG expression

SNCG expression was reviewed independently by two experienced pathologists (HF, PB). For external positive control (Figure 7. G-H) breast tumour tissue of a breast cancer patient was used. Immunohistochemical staining was considered positive, if a brown signal was cytoplasmatic or (peri)nuclear detectable.

## SUPPLEMENTARY FIGURES



## Abbreviations

AC: (extracellular) acidosis
BCSG 1: breast cancer-specific gene 1
bp: base pairs
BUB1: budding uninhibited by benzimidazoles 1
C: control
cDNA: complementary desoxy-ribonucleic acid
EC: endometrial cancer
EEC: endometroid endometrial carcinoma
ERα36: estrogen receptor alpha 36
ERK1/2: extracellular-signal regulated kinase 1/2
HE: hematoxylin and eosin staining
HX: hypoxia
JNK1: c-Jun N-terminal kinase 1
mRNA: messenger ribonucleic acid
NEEC: non-endometroid endometrial carcinoma
NMD: nonsense-mediated decay
ORF: open reading frame
PBS: phosphate-buffered saline
PCR: polymerase chain reaction
PTC: premature termination codon
SNCG: synuclein gamma
UTR: untranslated region
