# Supplementary material for: Alternative splicing of synuclein gamma in endometrial cancer: identification of a novel isoform
Source: Oncotarget. 2015 Jun 5;6(26):22553–63. doi: 10.18632/oncotarget.4155 (PMC4673181; doi:10.18632/oncotarget.4155)
Supplement: Supplementary file 1 [file oncotarget-06-22553-s001.pdf]

## SUPPLEMENTARY FIGURES

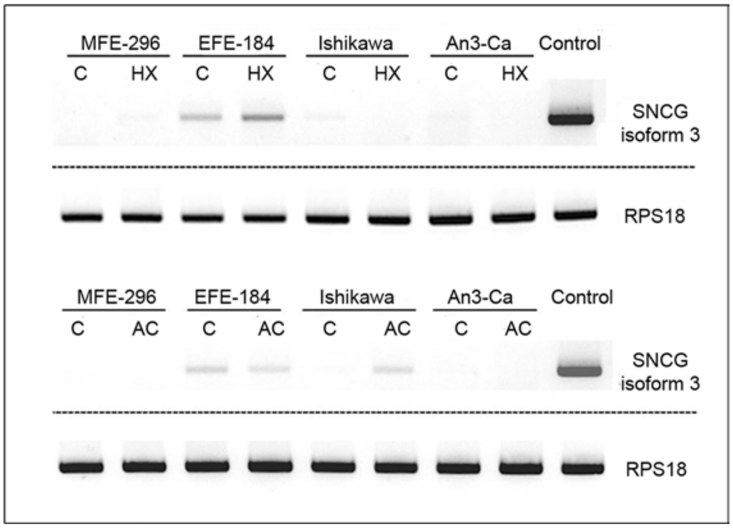


**Supplementary Figure S1: Expression of SNCG isoform 3 in EC cell lines MFE-296, EFE-184, Ishikawa, and An3-Ca under (C) control conditions, (HX) hypoxia (18 hrs, O2 > 1%) or (AC) acidosis (18 hrs, pH 6.2).** T47D breast cancer cells served as SNCG positive control. SNCG mRNA expression of isoform 3 is not or only marginal detectable in all tested EC cell lines compared to breast cancer cell line T47D. RPS18 expression serves as a comparative value. *Dashed lines indicate origin from different gels*. PCR.

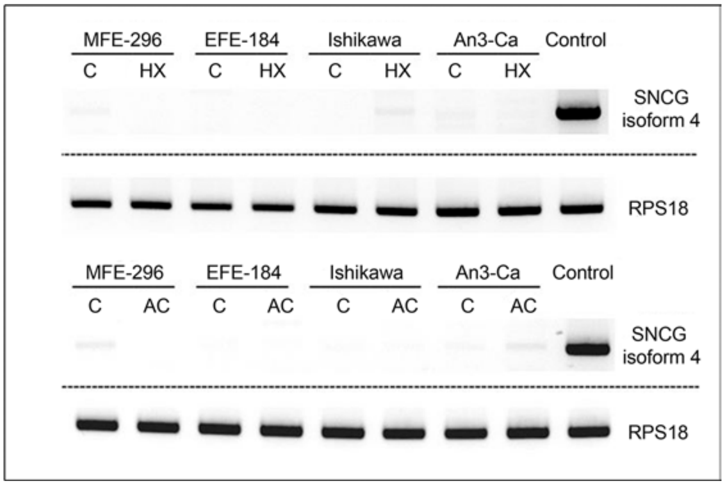


**Supplementary Figure S2: Expression of SNCG isoform 4 in EC cell lines MFE-296, EFE-184, Ishikawa, and An3-Ca under (C) control conditions, (HX) hypoxia (18 hrs, O2 > 1%) or (AC) acidosis (18 hrs, pH 6.2).** T47D breast cancer cells served as SNCG positive control. SNCG mRNA expression of isoform 4 is not or only marginal detectable in all tested EC cell lines compared to breast cancer cell line T47D. RPS18 expression serves as a comparative value. *Dashed lines indicate origin from different gels*. PCR.

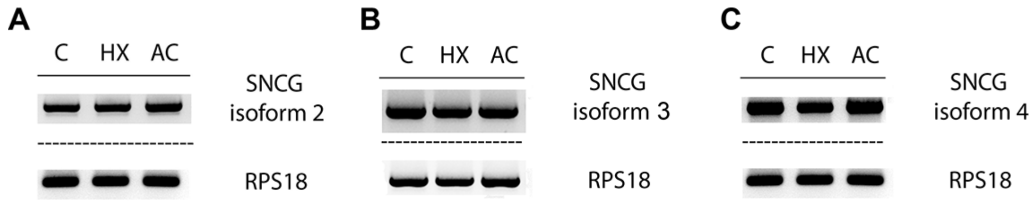


**Supplementary Figure S3: Expression of SNCG isoforms 2. A.** 3 **B.** and 4 **C.** in control cell line T47D under (C) control conditions, (HX) hypoxia (18 hrs, O2 > 1%) or (AC) acidosis (18 hrs, pH 6.2). Steady SNCG mRNA expression levels of all SNCG isoforms were detected under control conditions compared to hypoxia or extracellular acidosis. RPS18 expression serves as a comparative value. *Dashed lines indicate origin from different gels*. Figure displays one of triplicate experiments. PCR.
